# Prognostic factors of brain metastasis and survival among HER2-positive metastatic breast ﻿cancer patients: a systematic literature review

**DOI:** 10.1186/s12885-021-08708-5

**Published:** 2021-08-28

**Authors:** Michelle D. Hackshaw, Heather E. Danysh, Mackenzie Henderson, Eric Wang, Nora Tu, Zahidul Islam, Amy Ladner, Mary E. Ritchey, Maribel Salas

**Affiliations:** 1 Global Medical Affairs, Daiichi Sankyo, Basking Ridge, NJ USA; 2 Department of Epidemiology, RTI Health Solutions, Waltham, MA USA; 3 Global Epidemiology Department, Daiichi Sankyo, Inc., 211 Mt. Airy Road, Basking Ridge, NJ 07920 USA; 4 Rutgers Institute for Pharmaceutical Industry Fellowships, Piscataway, NJ USA; 5 Department of Epidemiology, RTI Health Solutions, Research Triangle Park, NC USA; 6 CCEB/CPeRT, University of Pennsylvania Perelman School of Medicine, Philadelphia, PA USA

**Keywords:** HER2-positive, HER2 +, Brain metastasis, Prognostic factors, Survival, Anti-HER2 therapy

## Abstract

**Background:**

Patients with breast cancer who overexpress the human epidermal growth factor receptor 2 (HER2) and subsequently develop brain metastasis (BM) typically experience poor quality of life and low survival. We conducted a comprehensive literature review to identify prognostic factors for BM and predictors of survival after developing BM, and the effects of therapies with different mechanisms of action among patients with HER2+ breast cancer (BC).

**Methods:**

A prespecified search strategy was used to identify research studies investigating BM in patients with HER2+ BC published in English during January 1, 2009–to June 25, 2021. Articles were screened using a two-phase process, and data from selected articles were extracted.

**Results:**

We identified 25 published articles including 4097 patients with HER2+ BC and BM. Prognostic factors associated with shorter time to BM diagnosis after initial BC diagnosis included younger age, hormone receptor negative status, larger tumor size or higher tumor grade, and lack of treatment with anti-HER2 therapy. Factors predictive of longer survival after BM included having fewer brain lesions (< 3 or a single lesion) and receipt of any treatment after BM, including radiosurgery, neurosurgery and/or systemic therapy. Patients receiving combination trastuzumab and lapatinib therapy or trastuzumab and pertuzumab therapy had the longest median survival compared with other therapies assessed in this review.

**Conclusions:**

More research is needed to better understand risk factors for BM and survival after BM in the context of HER2+ BC, as well as the assessment of new anti-HER2 therapy regimens that may provide additional therapeutic options for BM in these patients.

**Supplementary Information:**

The online version contains supplementary material available at 10.1186/s12885-021-08708-5.

## Background

Approximately 15 to 20% of patients with breast cancer (BC) have tumors with elevated levels of human epidermal growth factor receptor 2 (HER2), which are associated with an aggressive clinical phenotype and poor prognosis [1, 2]. Up to 50% of patients with HER2-positive (HER2+) metastatic BC will develop brain metastasis (BM) during the course of the disease, often leading to worse morbidity and shorter survival [3]. Current treatment strategies for BM in patients with HER2+ BC incorporate local therapies and systemic therapies. Local therapies include surgery, whole-brain radiotherapy (WBRT), and stereotactic radiosurgery [4–6]. Systemic therapies include chemotherapy (e.g., docetaxel, capecitabine) and anti-HER2 therapies, which can encompass monoclonal antibodies such as trastuzumab (approved in the U.S. in 1998 [7]) and pertuzumab (2012 [8]), antibody-drug conjugates such as trastuzumab emtansine (2013 [9]), and small molecule tyrosine kinase inhibitors (TKIs) such as lapatinib (2007 [10]) and neratinib (2017 [11]) [4–6]. Recent evidence suggests that lapatinib and neratinib can penetrate the blood-brain barrier (BBB), and therefore these drugs and similar HER2-targeting TKIs may be promising therapeutic options for patients [12, 13].

We conducted a literature review to assess the epidemiology of patients with HER2+ metastatic or advanced BC with BM by describing prognostic factors for developing BM and factors predictive of survival among patients with BM. Additionally, differences in survival and time to progression by HER2-targeting treatment drug classes were explored based on the drug mechanism of action.

## Methods

### Search design

Electronic searches were conducted in PubMed and Embase. A predefined search strategy (Online Resource 1 and Online Resource 2) was used to identify research studies investigating BM in patients with HER2+ BC. The search was restricted to studies published in English. The original search was restricted to studies published during the period of January 1, 2009 to July 30, 2019. However, the protocol was amended to expand the search to June 25, 2021 to capture the most recent published literature. Titles and abstracts identified from the electronic databases were exported to an Excel (Microsoft Corporation; Redmond, Washington) file for screening. Systematic literature reviews and meta-analyses relevant to the study objectives were not themselves included within the scope of this literature review, but the bibliographies were reviewed to identify potential additional publications.

### Screening and extraction

Articles were screened in a two-level process. In Level 1 screening, one researcher reviewed the titles and abstracts of the identified articles according to the literature review inclusion and exclusion criteria (Table 1) and selected articles for further review. In Level 2 screening, the full text of articles selected at Level 1 were reviewed by one researcher using the same set of inclusion and exclusion criteria. If there was any uncertainty about the inclusion of articles, the Level 2 reviewer discussed the article with a second researcher to confirm, by consensus, whether the article met the study inclusion and exclusion criteria. Data were extracted according to prespecified data fields using the full-text articles, including study population, country, observation period, sample size, and select sample characteristics. A checklist was used to assess the quality of the studies.

**Table 1:** Inclusion and Exclusion Criteria for Level 1 (Titles and Abstracts) and Level 2 (Full-Text) Screening

Criterion	Included	Excluded
Study design	▪ Randomized controlled trials ▪ Single-arm studies ▪ Observational research studies (e.g., prospective cohort study, retrospective database study, cohort study, case-control study) ▪ Literature reviews and meta-analyses^a^ ▪ Natural history studies ▪ Incidence and prevalence studies ▪ Prognostic factor studies	▪ Consensus reports ▪ Preclinical studies ▪ Nonsystematic reviews ▪ Case reports ▪ Case studies/series ▪ Editorials ▪ Commentaries ▪ Letters ▪ Guideline or position statements ▪ Economic analyses ▪ Animal or other nonhuman (e.g., bench) studies ▪ Study of < 25 patients
Population	▪ Patients with diagnosis of metastatic, recurrent, advanced, incurable, or unresectable HER2+ breast cancer (stages 3–4) with BM, either at the time of breast cancer diagnosis or after breast cancer diagnosis	▪ Aged under 18 y ▪ Only patients with HER2− or stage 1 or 2 breast cancer ▪ Only patients with HER2+ breast cancer without BM
Treatment	▪ Evaluation of chemotherapy by mechanism of action ▪ Nonpharmacological studies	▪ Specific regimens of chemotherapy (not mechanism of action) ▪ Patients receiving surgical or radiation intervention in place of chemotherapy ▪ Patients receiving CDK4/6 inhibitors
Evaluation	▪ Incidence or prevalence ▪ Prognostic and/or predictive factors ▪ Treatment outcomes (safety or effectiveness)	▪ PK/PD of treatments

*BM* brain metastasis; *CDK4/6* cyclin-dependent kinase 4 and 6; *HER2* human epidermal growth factor receptor 2; *PD* pharmacodynamics; *PK* pharmacokinetics

^a^ Literature reviews and meta-analyses were not be included in the review but were used to identify primary studies not previously identified

## Results

**Fig. 1:**
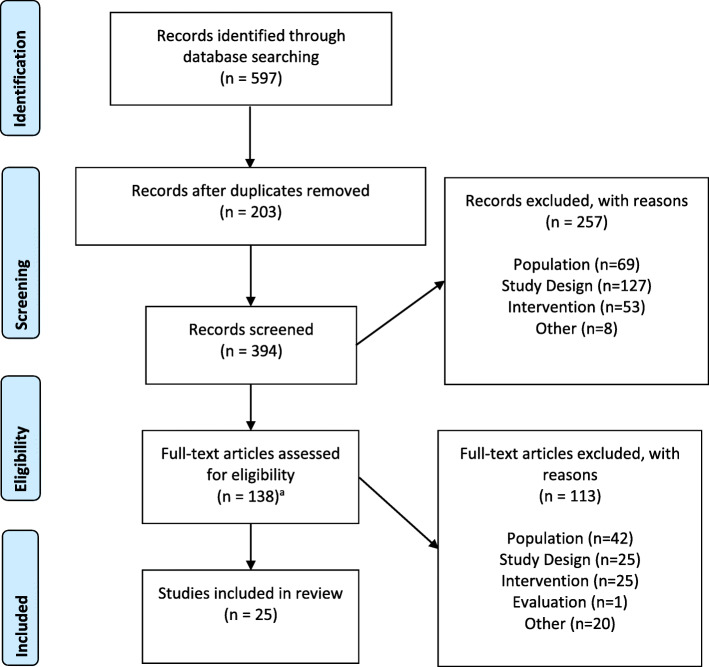
PRISMA Diagram. ^a^This includes one additional article identified from the review of the bibliography of a systematic review/meta-analysis after initial record screening, hence there appears to be one extra article in this diagram; PRISMA = Preferred Reporting Items for Systematic Reviews and Meta-Analyses

The number of studies included and excluded at each stage of screening was documented in a PRISMA diagram (Fig. 1) [14]. The original search strategy yielded 232 records for Level 1 screening and the expanded search added 162 records for Level 1 screening, for a total of 394 records retrieved. The bibliographies of 8 systematic reviews/meta-analyses were reviewed and yielded one additional study to be included for full-text screening for a total of 138 articles that were included in the Level 2 full-text screening. A total of 25articles met the inclusion criteria described in Table 1 and were selected for data extraction.

### Study and patient population characteristics

Online Resource 3 presents the study and patient characteristics of the 25studies included in this review. Overall, the studies covered more than 10,000 patients, of which 4097 patients had HER2+ BC with BM. Across all studies, the observation/enrollment period started as early as 1988 [15] and ended as late as 2020 [16]. Geographically, studies were conducted in Europe (*n* = 11), North America (*n* = 7), the Asia-Pacific region (*n* = 7), and Turkey (*n* = 1).

Among studies reporting the median age of patients at the time of their initial BC diagnosis (*n* = 19 [76%]), the median age range was 43 to 55 years. The sex distribution was typically not reported, although three studies did report including 100% females [17–19]. Only two studies reported on race, which included 67% white patients [15] and 58% white patients [20].

Twelve studies (48%) included only patients with HER2+ status [18, 20–28], while the remaining studies (*n* = 13 [52%]) reported on patients in whom a subset were HER2+, ranging from 10.1% [29] to 58.2% [30]. Among the seven studies that enrolled patients with or without BM [21–23, 26, 29, 31, 32], the prevalence of BM in patients with HER2+ BC ranged from 7.8% [29] to 56.0% [23]. Twenty-one studies (84%) provided information on hormone receptor (HR) status among patients with HER2+ BC, reporting a range of 24.2% [33] to 71.0% [26] of patients with HR+ status (i.e., estrogen receptor positive and/or progesterone receptor positive), while four studies (16%) did not report on HR status [16, 30, 34, 35].

### Prognostic factors for developing brain metastasis

Table 2 presents information on time to first BM diagnosis (TTBM) from initial HER2+ BC diagnosis and prognostic factors associated with a shorter TTBM. Across the 20 studies that reported information on TTBM, the shortest reported median TTBM was 10.8 months [31] and the longest was 76.2 months [26]. Among the 10 studies that reported on prognostic factors for BM diagnosis [15, 17, 18, 21–23, 26, 29–31], the most commonly assessed prognostic factors included age, HR status, receipt of anti-HER2 therapy, and tumor grade.

**Table 2:** Prognostic Factors Associated with Developing Brain Metastasis Among Patients with HER2+ Breast Cancer

Citation				Prognostic Factors for Shorter Time to BM				
	HER2+ Group	Sample Size, n	Median Time to BM^**a**^, mo	Age	HR Status	Anti-HER2 Therapy	Tumor Grade	Other
Ahn et al., 2013 [17]	Without trastuzumab	39	32.1	NR	NR	No association	NR	▪ NR
	With trastuzumab	47	35.4					
Anders et al., 2011 [15]	HR+	21	49.8 (95% CI, 10.2–54.5)	NR	HR- vs. HR+ (suggestive association)	NR	NR	▪ NR
	HR-	18	19.8 (95% CI, 13.6–36.2)					
Berghoff et al., 2012 [30]	All	102	18 (95% CI, 14.5–21.5)^b^	NR	ER- vs. ER+	No association	NR	▪ NR
	ER+	NR	NR	NR	NA	NR	NR	▪ Did not receive palliative endocrine therapy
Braccini et al., 2013 [36]	All	109	36 (range, 0–287)	NR	NR	NR	NR	▪ NR
Brufsky et al., 2011 [31]	All	377	10.8	< 50 y vs. ≥50 y	HR- vs. HR+	No trastuzumab vs. trastuzumab	NR	▪ ≥2 vs. < 2 metastatic sites
Duchnowska et al., 2012 [21]	All	142	13 (95% CI, 9–18)	No association	No association	NR	Tumor grade 3 vs. grade 1–2	▪ Higher H2T levels (≥50 RF/mm^2^)^c^ ▪ Time to nonbrain progression^d^ ▪ *HER-2* gene amplifications as defined by the *HER-2*/CEP17 ratio (no association) ▪ Menopausal status (no association)
Duchnowska et al., 2009 ^e^ [22]	All	264	15 (range, 0–81)^b^	No association	No association	No association	NR	▪ Time to distant relapse ≤2 y vs. > 2 y
Duchnowska et al., 2015 [23]	Cohort A (discovery)	83^f^	36 (range, 2–141)	NR	ER- vs. ER + ^e^	No trastuzumab vs. trastuzumab	No association	▪ Visceral site of first distant relapse ▪ 3-gene classifier^g^
	Cohort B (validation)	75	40 (range, 0.33–125)	NR	ER- vs. ER+	No trastuzumab vs. trastuzumab	Grade high vs. low ^e^	▪ Visceral site of first distant relapse
Gori et al., 2019 [24]	All	154	39.1 (IQR, 20.3–62.4)	NR	NR	NR	NR	▪ NR
Hayashi et al., 2015 [25]	All	432	33.5	NR	NR	NR	NR	▪ NR
Heitz et al., 2009 ^e^ [29]	All	245	30	No association	No association	No association	No association	▪ Pathological tumor size category 3/4 vs. category 1/2 ▪ TNM classification of metastatic (M) status at diagnosis is 1 vs. 0
Jang et al., 2011 [34]	All	137	31.6 (95% CI, 27.3–35.9)	NR	NR	NR	NR	▪ NR
Kuba et al., 2014 [35]	All	26	15.6 (range, 0–52.8)	NR	NR	NR	NR	▪ NR
Maurer et al., 2018 [26]	All	483	76.2	≤40 y vs. > 40 y	No association	No association	No association	▪ No surgery vs. surgery for primary BC ▪ Larger tumor size ▪ Nodal involvement ▪ Received adjuvant endocrine treatment ▪ Received no anthracyclines + taxanes as (neo) adjuvant chemotherapy
Morikawa et al., 2018 [27]	All	100	34.6 (range, 0–176)	NR	NR	NR	NR	▪ NR
Mounsey et al., 2018 [20]	All	123	34.6 (95% CI, 26.6–41.0)	NR	NR	NR	NR	▪ NR
Sperduto et al., 2013 [37]	HR+	98	47.4 (IQR, 26.3–70.5)	NR	NR	NR	NR	▪ NR
	HR-	119	35.8 (IQR, 13.4–69.2)	NR	NR	NR	NR	▪ NR
Witzel et al., 2018 [16]	All	732	32.4 (95% CI, 29.6–36.1)	NR	NR	NR	NR	▪ NR
Yap et al., 2012 [18]	All	280	30.1 (95% CI, 25.0–32.7)	NR	NR	No anti-HER2 treatment vs. anti-HER2 treatment	NR	▪ NR
Zhang et al., 2016 [28]	All	60	12 (range, 1–94)	NR	NR	NR	NR	▪ NR

*BC* breast cancer; *BM* brain metastasis; *CI* confidence interval; *ER* estrogen receptor; *H2T* the quantitative HER2 level as measured by the HERmark® Breast Cancer Assay; *HER-2/CEP17* HER-2/centromeric probe for chromosome 17 ratio > 2.0; *HR* hormone receptor; *IQR* interquartile range; *NA* not applicable; *NR* not reported; *RF* relative fluorescence; *TNM* TNM staging system (*T* tumor size and spread, *N* nodal involvement, M = metastatic status) developed by the American Joint Committee on Cancer

^a^ From the time of breast cancer diagnosis

^b^ From the time of diagnosis of metastatic disease

^c^ H2T is the quantitative HER-2 level as measured by the HERmark® Breast Cancer Assay (i.e., The VeraTag™ proximity-based assay; Monogram Biosciences, Inc., South San Francisco, California). The assay enables precise quantitative measurements of total HER-2 expression in formalin-fixed, paraffin-embedded tissue specimens. Higher H2T levels modeled as a continuous variable or as a categorical variable were associated with a shorter time to BM

^d^ Time from initiation of trastuzumab therapy to nonbrain progression. The direction of the effect was not specified in the article

^e^ Based on univariable analyses only

^f^ 83 of the 84 patient samples were analyzable

^g^ 3-gene classifier (including hepatoma-derived growth factor [HDGF], RAD51 homolog [RAD51], and translocated promoter region [TPR]) as a predictive model representing a 13-gene profile, which was associated with early (≤ 36 months) vs. late (> 36 months) BM and included the 3 genes in the 3-gene classifier and the following 11 genes: cyclin-dependent kinase 4 (*CDK4*), cyclin C (*CCNC*), focal adhesion kinase (protein tyrosine kinase 2, *PTK2*), v-myc avian myelocytomatosis viral oncogene homolog (*MYC*), breast cancer 1 [*BRCA1*] associated RING domain 1 (*BARD1*), Fanconi anemia group G (*FANCG*), proliferating cell nuclear antigen (*PCNA*), papillary renal cell carcinoma-translocation associated (*PRCC*), cortactin (*CTTN*), and desmoplakin (*DSP*)

In three studies, age was not found to be associated with TTBM [21, 22, 29], while two studies reported an association between younger age at BC diagnosis (aged ≤40 or <  50 years in Maurer et al. [26] and Brufsky et al. [31], respectively) and shorter TTBM [26, 31]. Hormone receptor status was assessed as a prognostic factor for TTBM in eight studies; four studies reported no association between HR status and TTBM [21, 22, 26, 29], while four other studies reported that patients with HR- status had a shorter TTBM compared with those who were HR+ [15, 23, 30, 31]. Among the eight studies that assessed trastuzumab use and TTBM, five reported no association between receipt of trastuzumab and TTBM [17, 22, 26, 29, 30], while three reported that patients who received trastuzumab had a longer TTBM compared with those who did not [18, 23, 31]. Two studies reported an association between higher tumor grade and shorter TTBM [21, 23], while two studies reported no association with tumor grade but did report an association between larger tumor size and shorter TTBM [26, 29].

Other treatment-related factors and TTBM were also assessed. In one study, longer TTBM was reported among those receiving treatment with endocrine therapy versus no endocrine therapy in the palliative care setting [30]. Conversely, another study reported that patients receiving adjuvant endocrine treatment had a shorter TTBM [26]. Longer TTBM was reported among patients who had surgery for their BC and among those treated with (neo) adjuvant anthracyclines + taxanes [26].

Other reported prognostic factors for shorter TTBM included higher disease burden (i.e., ≥ 2 metastatic sites) [31], higher HER2 levels (measured by the HERmark® Breast Cancer Assay) [21], distant relapse in ≤2 years [22], first distant relapse at a visceral site [23], and nodal involvement [26]. One study reported on genetic factors of TTBM, but the reported associations in the discovery set were not observed in the validation set [23].

### Predictors of survival after brain metastasis among patients with HER2+ breast cancer

Table 3 presents median time to death (i.e., survival) after BM diagnosis and predictive factors associated with shorter survival after BM diagnosis. Across the 23 studies that reported information on median survival after BM, the shortest reported median survival was 5.2 months [34] and the longest was 28 months [21].

**Table 3:** Predictors of Survival Among Patients with HER2+ Breast Cancer and Brain Metastasis

Citation				Predictors for Shorter Time to Death (i.e., Survival) After BM Diagnosis					
	HER2+ Group	Sample Size, n	Median Time to Death After BM Diagnosis, mo	Age	HR Status	No. of Brain Lesions	Anti-HER2 Therapy	Any Systemic Treatment	Other
Ahn et al., 2013 [17]	Without trastuzumab	39	19.1^a^	NR	NR	NR	No trastuzumab vs. trastuzumab	NR	NR
	With trastuzumab	47	26.9^a^						
Anders et al., 2011 [15]	HR-	18	14.3 (95% CI, 3.2–36.2)	No association	No association	NR	NR	NR	Race (no association)
	HR+	21	15.2 (95% CI, 7.8–40.4)						
Anwar et al., 2021 [32]	All	39	13.93 (95% CI 10.53–20.67)^b^	NR	No association	NR	NR	NR	▪ Number of prior lines of therapy (no association) ▪ Liver metastasis (no association) ▪ Lung metastasis (no association) ▪ Bone metastasis (no association)
Bergen et al., 2021 [19]	All	252	Before 2000: 12 2000–2010: 11 After 2010: 22	Included in DS-GPA but not reported independently	Included in DS-GPA but not reported independently	NR	▪ No HER2-targeted therapy vs. therapy with trastuzumab + pertuzumab, or trastuzumab alone, or lapatinib alone, or T-DM1 alone ▪ Other HER2-targeted therapy vs. trastuzumab + pertuzumab ▪ No HER2-targeted therapy vs. trastuzumab + lapatinib (no association)	NR	▪ DS-GPA ▪ Time period (year) of initial BC diagnosis (< 2000, 2000–2010, > 2010) (no association)
Berghoff et al., 2012 [30]	All	102	7 (95% CI, 4.3–969)	NR	NR	NR	▪ Local therapy^c^ alone vs. trastuzumab-based therapy after local therapy	NR	NR
Braccini et al., 2013 [36]	All	109	11.9 (95% CI, 8.7–15.5)	NR	No association	NR	▪ No anti-HER2 therapy vs. anti-HER2 therapy ▪ Trastuzumab alone or lapatinib alone vs. trastuzumab + lapatinib (sequentially)	NR	NR
Brufsky et al., 2011 [31]	All	377	13.0 (range, 0.1–55.5)^d^	No association	No association	NR	▪ No trastuzumab vs. trastuzumab	▪ No chemotherapy vs. chemotherapy	▪ No surgery vs. surgery ▪ Radiotherapy – no association ▪ ECOG PS ≥ 2 vs. 0 or 1 ▪ CNS disease at mBC diagnosis vs. no CNS disease at mBC diagnosis
Duchnowska et al., 2012 [21]	All	142	28 (95% CI, 16–32)	NR	NR	NR	▪ NR	NR	NR
Gori et al., 2019 [24]	All	154	24.5	≥ 60 y vs. < 60 y at BM diagnosis^e^	No association	> 3 vs. 1–3 BMs^e^	▪ Systemic therapy without HER2-targeted agents or no systemic therapy vs. HER2-targeted agents	NR	▪ WBRT or no local treatment vs. surgery and/or SRS ▪ KPS ≤ 7 0 vs. > 70 ▪ Presence of neurologic symptoms
Hayashi et al., 2015 [25]	ER+	162	16.5 (95% CI, 11.9–21.1)	NR	No association	> 3 vs. ≤ 3 BMs	▪ Neither trastuzumab nor lapatinib vs. at least one of these after BM diagnosis ▪ Either trastuzumab alone, lapatinib alone, or no HER2-targeting agent vs. trastuzumab and lapatinib after BM diagnosis	NR	NR
	ER-	270	11.5 (95% CI, 9.1–13.8)						
Heitz et al., 2009 ^f^ [29]	All	245	11	NR	NR	NR	▪ NR	NR	▪ NR
Jang et al., 2011 [34]	All	137	5.2 (95% CI, 3.6–6.8)	NR	NR	NR	▪ NR	NR	▪ NR
Kaplan et al., 2012 [33]	ER−/PR-	102	11.04 (95% CI, 6.18–15.90)	≥ 46 y vs. < 46 y at BM diagnosis^e^ (suggestive association in multivariable analyses)	No association	> 3 vs. ≤ 3 BMs^e^ (suggestive association in multivariable analyses)	▪ Trastuzumab- or lapatinib-based therapy alone vs. trastuzumab- and lapatinib-based therapy (sequential) ▪ Trastuzumab-based therapy alone vs. lapatinib-based therapy alone	NR	▪ KPS ≤ 70 vs. > 70 ▪ Tumor grade 3 vs. grade 1–2 ▪ ≥ 2 vs. < 2 metastatic sites outside the brain ▪ No neurosurgery vs. neurosurgery ▪ No radiosurgery vs. radiosurgery
	Luminal B^f^	113	9.99 (95% CI, 4.99–14.98)						
Kuba et al., 2014 [35]	All	26	23 (95% CI, 14–31)	No association	NR	No association	▪ NR	NR	▪ PS ≥ 2 vs. 0/1 ▪ Undergoing surgery or SRS (no association)
Martin et al., 2017 [38]	HR+	136	21 (IQR: 6-not reached)^g^	NR	NR	NR	▪ NR	NR	▪ NR
	HR-	106	10 (IQR: 4–27)^g^						
Maurer et al., 2018 [26]	All	483	20.8 (IQR: 5.36-not reached)	NR	NR	NR	▪ No association	NR	▪ CNS symptoms^h^ vs. no CNS symptoms at BM diagnosis
Morikawa et al., 2018 [27]	All	100	19.4 (95% CI, 15.5–26.6)	No association	No association	Multiple lesions vs. single lesion	▪ No anti-HER2 use vs. anti-HER2 use after BM diagnosis ▪ No anti-HER2 use vs. lapatinib use after BM diagnosis	NR	▪ KPS < 70 vs. ≥ 70 ▪ Neurologic symptoms vs. no neurologic symptoms ▪ Uncontrolled extracranial disease vs. controlled
Mounsey et al., 2018 [20]	All	123	18.1 (95% CI, 14.9–24.6)	NR	NR	NR	▪ No HER2-targeted therapy vs. HER2-targeted therapy after BM diagnosis	NR	▪ NR
Niwinska et al., 2010 [39]	All	109	9 (range, 0.6–3.4)	NR	NR	NR	▪ No systemic therapy or chemotherapy without trastuzumab vs. chemotherapy with trastuzumab	WBRT alone vs. systemic therapy^i^ after WBRT	▪ KPS < 70 vs. ≥ 70 ▪ RPA RTOG Prognostic class III vs. class I/II ▪ Visceral metastasis vs. no visceral metastasis
Sperduto et al., 2013 [37]	HR+	98	22.9 (95% CI, 16.1–29.5)	NR	NR	NR	▪ NR	NR	▪ NR
	HR-	119	17.9 (95% CI, 13.4–22.9)						
Witzel et al., 2018 [16]	All	732	11.6 (95% CI, 10.0–13.4)	NR	NR	NR	▪ NR	NR	▪ NR
Yap et al., 2012 [18]	All	280	10.9 (95% CI, 9.0–11.9)	Older age vs. younger age at BM	NR	Multiple lesions vs. single lesion	▪ No anti-HER2 treatment vs. anti-HER2 treatment after BM diagnosis ▪ No anti-HER2 treatment or trastuzumab alone vs. lapatinib alone after BM diagnosis ▪ No anti-HER2 treatment vs. trastuzumab alone after BM diagnosis ▪ Anti-HER2 therapy before BM (no association with survival after BM)	▪ No chemotherapy vs. receipt of chemotherapy after BM diagnosis ▪ No hormonal therapy vs. receipt of hormonal therapy after BM	▪ NR
Zhang et al., 2016 [28]	All	60	12 (range, 1–94)	< 50 y vs. ≥ 50 y at BM diagnosis^e^	No association	Multiple lesions vs. single lesion^e^	▪ No anti-HER2 therapy after WBRT vs. anti-HER2 therapy after WBRT ▪ No systemic therapy, anti-HER2 therapy alone, or chemotherapy alone after WBRT vs. both anti-HER2 therapy and chemotherapy after WBRT	[See “Anti-HER2 therapy” column]	▪ Uncontrolled extracranial metastasis vs. controlled ▪ KPS < 70 vs. ≥ 70^e^ ▪ Total dose radiotherapy (no association)^e^ ▪ Time from BC diagnosis to BM diagnosis (no association)^e^

*BC* breast cancer; *BM* brain metastasis; *CI* confidence interval; *CNS* central nervous system; *DS-GPA* diagnosis specific graded prognostic assessment

Score (includes BC subtype, age < 60 or > 60 years, Karnofsky performance status), *ECOG* Eastern Cooperative Oncology Group; *ER* estrogen receptor; *HER2* human epidermal growth factor receptor 2; *HR* hormone receptor; *IQR* interquartile range; *KPS* Karnofsky performance score; *mBC* metastatic breast cancer; *NR* not reported; *PR* progesterone receptor; *PS* performance status; *RPA RTOG* recursive partitioning analysis of Radiation Therapy Oncology Group prognostic class; *SRS* stereotactic radiosurgery; *T-DM1* trastuzumab emtansine; *WBRT* whole-brain radiotherapy

^a^ Time from diagnosis of distant metastasis

^b^ Represents median time to death after start of pyrotinib therapy

^c^ Local therapy for BM including surgery and/or radiotherapy

^d^ Overall survival after BM for all patients diagnosed with BM, including patients who presented with BM at the time of their mBC diagnosis (*n* = 75 [19.9%]; overall survival after diagnosis was 20.3 months [range, 1.0–55.5]) and patients who were diagnosed with BM after their mBC diagnosis (*n* = 302 [80.1%]; overall survival after BM diagnosis was 9.6 months [range, 0.1–54.5])

^e^ Based on univariable analyses only

^f^ Luminal B subtype is defined as HER2+ status with ER+ and/or PR+

^g^ Survival defined as the time between BC diagnosis and death

^h^ The most common symptoms were headaches (50.0%), nausea and vomiting (25.0%), confusion and memory impairment (18.2%), paresis (18.2%), aphasia and dysarthria (6.8%), and seizures (6.8%)

^i^ Includes chemotherapy, endocrine therapy, and HER2-targeted therapy

The most commonly assessed predictive factors for shorter survival after BM diagnosis included age, HR status, number of brain lesions, receipt of anti-HER2 therapy, and receipt of any systemic therapy. Four studies reported no association between age at BM diagnosis and survival [15, 27, 31, 35], three reported shorter survival among older patients [18, 24, 33], and one reported shorter survival among younger patients [28]. The nine studies that observed HR status reported no association between HR status and survival after BM diagnosis [15, 24, 25, 27, 28, 31–33, 36]. Six studies reported that the presence of a higher number brain lesions compared with fewer brain lesions was associated with shorter survival after BM diagnosis [18, 24, 25, 27, 28, 33]. Other reported predictors of shorter survival after BM diagnosis included the presence of neurologic symptoms [24, 26, 27], tumor grade 3, two or more extracranial metastatic sites [33], central nervous system disease at BC diagnosis [31], uncontrolled extracranial metastases [27, 28], visceral metastases, and Radiation Therapy Oncology Group recursive partitioning analysis prognostic class 3 versus class 1/2 [39].

While specific regimens were not assessed in this literature review, data were included from studies that did not assign treatment and for which any anti-HER2 treatment was captured within typical clinical practice. All 13 studies that assessed treatment with anti-HER2 therapy and survival after BM diagnosis reported an association between anti-HER2 therapy and survival. Twelve studies reported that patients who received anti-HER2 therapy after their BM diagnosis had a longer survival compared with patients who did not receive anti-HER2 therapy [17–20, 24, 25, 27, 28, 30, 31, 36, 39]. Four studies [18, 25, 33, 36] found that patients receiving both trastuzumab and lapatinib after their BM diagnosis had longer survival than those receiving either agent alone or no anti-HER2 therapy. One study found that patients receiving trastuzumab and pertuzumab after their BM diagnosis had longer survival than those receiving other HER2-targeted therapies or no HER2-targeted therapy [19].

Four studies [18, 31, 35, 39] assessed non-HER2-targeted therapies and survival after BM diagnosis and reported that shorter survival was associated with no chemotherapy versus chemotherapy [18, 31], no hormonal therapy versus hormonal therapy [18], WBRT alone versus any systemic therapy after WBRT [39], and no systemic therapy versus any systemic therapy [35]. In addition, three studies reported that patients receiving surgery or stereotactic radiosurgery had longer survival than those not receiving these treatments [24, 31, 33], while one study reported no association [35]. One study reported no association between the total dose of radiotherapy and survival after BM diagnosis [28].

### Treatment mechanism of action and outcomes after brain metastasis

#### HER2-targeted monoclonal antibodies

Table 4 presents information on anti-HER2 treatment type by mechanism of action, and disease progression and survival after BM. Four studies evaluated survival among patients receiving a HER2-targeted monoclonal antibody (i.e., trastuzumab) after BM diagnosis and reported that overall survival (OS) was longer in patients who received trastuzumab after local therapy compared with patients who did not receive trastuzumab [17, 30, 31, 39]. In Ahn et al. [17], Berghoff et al. [30], and Brufsky et al. [31], the difference in median OS after BM between trastuzumab users and nonusers was 7.8 months, 10 months, and 13.8 months, respectively. Niwinska et al. [39] reported that among patients with HR+ status, trastuzumab users had a 11-month longer median OS compared with nonusers (*P* < 0.001), and among patients with HR- status, trastuzumab users had a 6-month longer median OS compared with nonusers (*P* = 0.004). One study evaluated survival among patients receiving HER2-targeted monoclonal antibody combination therapy (i.e., trastuzumab + pertuzumab) and reported that OS was longer in patients who received trastuzumab + pertuzumab (44 months) compared to those who received other HER2-targeted therapy (17 months) or no HER2-targeted therapy (3 months) [19].

**Table 4:** Effect of Treatment Mechanisms of Action on Survival, Tumor Response, Time to Progression

Citation	Therapy After BM	Outcome Assessed
**HER2-targeted monoclonal antibodies**		
Ahn et al., 2013 [17]	Trastuzumab	OS after diagnosis of distant metastasis: trastuzumab, 26.9 mo; no trastuzumab, 19.1 mo; *P* = 0.020
Berghoff et al., 2012 [30]	Trastuzumab	OS after BM diagnosis, 7 mo (95% CI, 4.3–969); trastuzumab-based therapy after completion of local therapy for BM (surgery, radiotherapy), 14 mo (95% CI, 7.22–20.78); vs. not, 4 mo (95% CI, 2.40–5.61)
Brufsky et al., 2011 [31]	Trastuzumab	OS after BM diagnosis, 13.0 mo (range, 0.1–55.5); trastuzumab^a^, 17.5 mo; vs. no trastuzumab, 3.7 mo; adjusted hazard ratio = 0.33 (95% CI, 0.25–0.46)
Niwinska et al., 2010 [39]	Trastuzumab	OS after BM diagnosis ▪ HER2+/HR+: trastuzumab + chemotherapy after WBRT, 13 mo; chemotherapy alone after WBRT, 8 mo; no systemic treatment after WBRT, 2 mo; *P* < 0.001 ▪ HER2+/HR-: trastuzumab + chemotherapy after WBRT, 10 mo; chemotherapy alone after WBRT, 8 mo; no systemic treatment after WBRT, 4 mo; *P* = 0.004
Bergen et al., 2021 [19]	Trastuzumab + Pertuzumab	OS after BM diagnosis, trastuzumab + pertuzumab, 44 mo; other HER2-targeted therapy, 17 mo; no HER2-targeted therapy, 3 mo (*P* < 0.001) Overall intracranial CBR, trastuzumab + pertuzumab as systemic first-line therapy after diagnosis of BM, 100% Overall intracranial ORR, trastuzumab + pertuzumab as systemic first-line therapy after diagnosis of BM, 92.9%
**HER2-targeted non-monoclonal antibodies**		
Anwar et al., 2021 [32]	Pyrotinib	OS after pyrotinib initiation, pyrotinib + surgery/radiation, 20.7 mo; pyrotinib only, 12.4 mo (*P* = 0.021) PFS after pyrotinib initiation, pyrotinib + surgery/radiation, 10.0 mo; pyrotinib only, 7.7 mo (*P* = 0.19) CBR after pyrotinib initiation, pyrotinib + surgery/radiation, 58.6%; pyrotinib only, 41.4% ORR after pyrotinib initiation pyrotinib + surgery/radiation, 24.1%; pyrotinib only, 31.0%
**HER2-targeted monoclonal antibodies + Tyrosine kinase inhibitors**		
Braccini et al., 2013 [36]	Trastuzumab + Lapatinib	OS after BM diagnosis, trastuzumab and lapatinib (sequential), 25.7 mo (95% CI, 17.1–33.3); only 1 of the 2 targeted therapies, 9.6 mo (95% CI, 8.2–12.8); *P* < 0.001
Kaplan et al., 2012 [33]	Trastuzumab + Lapatinib	OS after BM diagnosis, trastuzumab- and lapatinib-based therapy (sequential), 23.6 mo; only 1 of the 2 targeted therapies, 14.6 mo; *P* = 0.023
Hayashi et al., 2015 [25]	Trastuzumab + Lapatinib	OS after BM diagnosis, trastuzumab and lapatinib had a longer survival^b^ than trastuzumab alone, lapatinib alone, or no HER2-targeting agent; *P* < 0.001
**Tyrosine kinase inhibitors + other anti-HER2 therapies (not otherwise specified)**		
Morikawa et al., 2018 [27]	Anti-HER2 therapy + Lapatinib	OS from BM diagnosis, 19.4 mo (95% CI, 15.5–26.6); anti-HER2 therapy with lapatinib vs. no use, adjusted hazard ratio = 0.26 (95% CI, 0.13–0.52); anti-HER2 therapy without lapatinib vs. no use, adjusted hazard ratio = 0.32 (95% CI, 0.18–0.59)
**Anti-HER2 therapies (not otherwise specified)**		
Braccini et al., 2013 [36]	Anti-HER2 therapy	▪ OS after BM diagnosis, 11.9 mo (95% CI, 8.7–15.5); anti-HER2 therapy, 15.2 mo (95% CI, 11.5–19.4); without anti-HER2 therapy, 3.4 mo (95% CI, 1.4–6) ▪ Cerebral progression-free survival, anti-HER2 therapy, 6.3 mo (95% CI, 7.8–11.5); without anti-HER2 therapy, 5.5 mo (95% CI, 1.2–6.7)
Kaplan et al., 2012 [33]	HER2-targeted therapy (includes all patients receiving trastuzumab, lapatinib, or both)	OS after BM diagnosis; HER2-targeted therapy, 16.7 mo; without HER2-targeted therapy, 4.7 mo; *P* < 0.001
Gori et al., 2019 [24]	Anti-HER2 therapy	OS after BM diagnosis, 24.5 mo; HER2-targeted therapy (27.5 mo) vs. without anti-HER2 therapy (13.8 mo) (hazard ratio = 0.44 [95% CI, 0.25–0.78]) vs. no systemic therapy (2.1 mo) (hazard ratio = 0.09 [95% CI, 0.05–0.16])
Maurer et al., 2018 [26]	Anti-HER2 treatment	No impact on the development of a second CNS event or on OS. OS, 20.8 mo (IQR, 5.36-not reached)
Mounsey et al., 2018 [20]	HER2-targeted therapy (includes trastuzumab, lapatinib, pertuzumab, and T-DM1)	▪ Mortality after BM, receipt of HER2-targeted therapy after BM diagnosis, adjusted hazard ratio = 0.61 (95% CI, 0.39–0.97) ▪ OS after BM diagnosis, 18.1 mo (95% CI, 14.9–24.6); HER2-targed therapy (62% of patients), 25.3 mo (95% CI, 18.6–31.2); without HER2-targeted therapy, 7.8 mo (95% CI, 4.56–15.0)
Yap et al., 2012 [18]	Anti-HER2 therapy (includes trastuzumab alone, lapatinib alone, or trastuzumab and lapatinib combined)	OS after BM diagnosis, 10.9 mo (95% CI, 9.0–11.9); anti-HER2 therapy, 18.5 mo; no anti-HER2 therapy, 5.7 mo; adjusted hazard ratio = 0.62 (95% CI, 0.43–0.89)
Zhang et al., 2016 [28]	Anti-HER2 therapy (includes trastuzumab alone, lapatinib alone, or trastuzumab and lapatinib combined)	OS after BM diagnosis, 12 mo (range, 1–94); anti-HER2 therapy after WBRT, 21 mo, no anti-HER2 therapy after WBRT, 9 mo; *P* = 0.002
Bergen et al., 2021 [19]	HER2-targeted therapy, or no HER2-targeted therapy	OS after BM diagnosis, other HER2-targeted therapy, 17 mo; no HER2-targeted therapy, 3 mo

*BM* brain metastasis; *CBR* clinical benefit rate; *CI* confidence interval; *CNS* central nervous system; *HER2* Human Epidermal Growth Factor 2; *HR* hormone receptor; *IQR* interquartile range; *ORR* overall response rate; *OS* overall survival; *T-DM1* ado-trastuzumab emtansine; *WBRT* whole-brain radiotherapy

^a^ 27.5% of patients who received trastuzumab after BM diagnosis also received lapatinib (mostly after trastuzumab). No patients received only lapatinib after BM diagnosis

^b^ Survival months not reported

#### HER2-targeted non-monoclonal antibodies

One study evaluated survival among patients receiving a HER2-targeted non-monoclonal antibody (i.e., pyrotinib) with or without surgery/radiation, and reported that OS was longer in those with combination HER2-targeted non-monoclonal antibody and surgery/radiation [32]. OS was 20.7 months in those that received both a HER2-targeted non-monoclonal antibody (i.e., pyrotinib) and surgery/radiation, compared with 12.4 months in those who received only a HER2-targed non-monoclonal antibody (i.e., pyrotinib) [*P* = 0.021] [32].

#### Tyrosine kinase inhibitor combination therapies

Four studies evaluated the association between patients receiving lapatinib in combination with either trastuzumab or another anti-HER2 therapy, not otherwise specified [25, 27, 33, 36]. Braccini et al. [36] and Kaplan et al. [33] reported that patients receiving both lapatinib and trastuzumab had longer median OS (25.7 months and 23.6 months, respectively) after BM compared with those receiving either lapatinib alone or trastuzumab alone (9.6 months [*P* < 0.001] and 14.6 months [*P* = 0.023], respectively). Hayashi et al. [25] reported longer OS after BM in patients receiving lapatinib and trastuzumab compared with patients receiving only one of the two targeted therapies or no HER2-targeting therapy (*P* < 0.001). Similarly, Morikawa et al. [27] reported that patients receiving lapatinib in combination with another anti-HER2 therapy, not otherwise specified, had lower mortality compared with those not receiving the combination therapy (adjusted hazard ratio = 0.26; 95% confidence interval [CI], 0.13–0.52).

#### Any anti-HER2 therapy

The use of anti-HER2 therapy, not otherwise specified, after BM diagnosis was associated with an increase in OS in all studies except one [26]. Among the seven studies that reported longer survival in patients treated with anti-HER2 therapy after BM diagnosis [18–20, 24, 28, 33, 36], the median OS among those receiving anti-HER2 therapy ranged from 15.2 to 44 months compared with the median OS among those not receiving anti-HER2 therapy, which ranged from 3 to 13.8 months. Among patients receiving anti-HER2 therapy after BM diagnosis, median OS ranged from 11.8 [36] to 17.5 months [20] longer after their BM diagnosis compared with those not receiving anti-HER2 therapy. Conversely, Maurer et al. [26] reported no association between anti-HER2 therapy after BM diagnosis and OS.

Two studies reported on cerebral disease progression after BM diagnosis and treatment with anti-HER2 therapy [26, 36]. Braccini et al. [36] reported longer cerebral progression-free survival in patients treated with anti-HER2 therapy than in patients not receiving anti-HER2 therapy (6.3 months [95% CI, 7.8–11.5] vs. 5.5 months [95% CI, 1.2–6.7]), while Maurer et al. [26] reported no association between anti-HER2 therapy and a second central nervous system event.

#### Quality of studies

Quality assessment of included studies was conducted using the Good Research for Comparative Effectiveness (GRACE) checklist [40, 41]. This 11-item scale contains 6 items related to quality of data and 5 items related to methodology. For each question, the quality is assessed based on “fit for purpose”, and the quality is considered sufficient if the data or information provided per item is adequate for study purposes. The quality assessment revealed that all studies were eligible to be included in this review, even though 5 (20%) studies have some limitations in the scientific methods (See Table 5).

**Table 5:** Quality of Studies Included

Citation	Data Quality ^**a**^	Scientific Method ^**b**^	Overall Quality Rating
Anders et al., 2011 [15]	Sufficient	Sufficient	Sufficient
Witzel et al., 2018 [16]	Sufficient	Sufficient	Sufficient
Ahn et al., 2013 [17]	Sufficient	Insufficient	Sufficient
Yap et al., 2012 [18]	Sufficient	Sufficient	Sufficient
Mounsey et al., 2018 [20]	Sufficient	Sufficient	Sufficient
Duchnowska et al., 2012 [21]	Sufficient	Insufficient	Sufficient
Duchnowska et al., 2009 [22]	Sufficient	Insufficient	Sufficient
Duchnowska et al., 2015 [23]	Sufficient	Sufficient	Sufficient
Gori et al., 2019 [24]	Sufficient	Sufficient	Sufficient
Hayashi et al., 2015 [25]	Sufficient	Sufficient	Sufficient
Maurer et al., 2018 [26]	Sufficient	Sufficient	Sufficient
Morikawa et al., 2018 [27]	Sufficient	Sufficient	Sufficient
Zhang et al., 2016 [28]	Sufficient	Sufficient	Sufficient
Heitz et al., 2009 ^e^ [29]	Sufficient	Sufficient	Sufficient
Berghoff et al., 2012 [30]	Sufficient	Sufficient	Sufficient
Brufsky et al., 2011 [31]	Sufficient	Sufficient	Sufficient
Kaplan et al., 2012 [33]	Sufficient	Sufficient	Sufficient
Jang et al., 2011 [34]	Sufficient	Insufficient	Sufficient
Kuba et al., 2014 [35]	Sufficient	Sufficient	Sufficient
Braccini et al., 2013 [36]	Sufficient	Sufficient	Sufficient
Niwinska et al., 2010 [39]	Sufficient	Sufficient	Sufficient
Sperduto et al., 2013 [37]	Sufficient	Insufficient	Sufficient
Martin et al., 2017 [38]	Insufficient	Sufficient	Sufficient
Anwar et al., 2021 [32]	Sufficient	Sufficient	Sufficient
Bergen et al., 2021 [19]	Sufficient	Sufficient	Sufficient

a - Data attributes of exposure, outcomes (recording, objective measurement, validation) and important covariates/co-founders were assessed using 6 item checklist

b – Scientific methods were assessed using 5 item checklist and focused on the following areas

- New initiators of treatment

- Concurrent comparators

- Control of covariates/confounders/effect modifier

- Control of immortal time bias

- Analyses to evaluate the potential for bias for biased assessment

## Discussion

This literature review included 25 published articles that assessed a total of 4097patients with HER2+ BC with BM. Among these patients, prognostic factors of BM development and predictive factors of survival after BM diagnosis were assessed. Prognostic factors associated with shorter TTBM among patients with HER2+ BC included younger age at BC diagnosis, HR- versus HR+ status [15, 23, 30, 31], no receipt of trastuzumab versus receipt of trastuzumab [18, 23, 31], and higher tumor grade versus lower grades [21, 23]. While these associations were observed across multiple studies, six studies found no association with TTBM and these prognostic factors [17, 21, 22, 26, 29, 30]. Some studies reported longer TTBM in patients receiving trastuzumab or anti-HER2 therapy, not otherwise specified, while some studies found no association.

Overall survival after BM diagnosis was shorter in patients with a higher number of lesions, was unaffected by HR status, and was variably affected by age at diagnosis. Treatment-related factors predictive of longer survival after BM included receipt of any systemic therapy. Studies that assessed treatment with anti-HER2 therapy after BM diagnosis and survival (*n* = 13) reported that patients who received anti-HER2 therapy had longer survival after BM compared with patients who did not receive anti-HER2 therapy. Among studies that assessed survival differences between trastuzumab-based and lapatinib-based therapies [18, 25, 33, 36], patients receiving both trastuzumab and lapatinib after BM diagnosis had longer survival than those receiving either agent alone or no anti-HER2 therapy. One study found that patients receiving both trastuzumab and pertuzumab therapy after BM diagnosis had longer survival than those receiving other HER2-targeted therapy or no HER2-targeted therapy [19]. While trastuzumab has limited capability to cross the intact BBB, elevated concentrations of trastuzumab in the cerebrospinal fluid have been demonstrated when the BBB is impaired via radiotherapy and/or meningeal carcinomatosis [42]. As described in this review, trastuzumab in combination with lapatinib, which has been shown to cross the BBB in the BM setting [43], is favorable for survival in patients with BM. Lapatinib-based therapies may be an alternative therapeutic option for patients with BM and trastuzumab resistance [44]. The studies in this review also reported that survival after BM was improved with other therapies, including surgery or radiosurgery [24, 31, 33] and other systemic therapies [18, 31, 39]; however, the improvements in survival in patients receiving anti-HER2 therapy superseded survival in those receiving one of these other therapies alone [24, 28, 39].

Contemporary patients with HER2+ BC have better OS compared with patients with HER2+ status who received treatment for BC 20 years ago [45]. As more patients with HER2+ BC are living longer, with more opportunity to develop distant metastases, new investigational agents are needed to treat patients with HER2+ BC with BM. One such drug is tucatinib, a next-generation small molecule TKI that is currently under assessment for improving outcomes specifically among patients with HER2+ BC with BM (ClinicalTrials.gov: NCT02614794 and NCT03975647) [46]. A recent randomized controlled trial found the addition of tucatinib compared with placebo to trastuzumab and capecitabine regimens improved progression-free survival and OS [47]. Trastuzumab deruxtecan, an antibody-drug conjugate, demonstrated encouraging antitumor activity during a phase 2 trial that evaluated patients with HER2+ metastatic BC, including 24 patients with BM [48]. Neratinib, a pan-HER TKI, showed efficacy in combination with capecitabine for treatment of refractory HER2+ BC with BM [49]. Treatment with anti-HER2 therapies, including current investigational and newly approved therapies, may provide additional options for patients with HER2+ BC with BM.

This review includes some limitations, such as the search was limited to the past 10 years and to English-language articles only. Only studies that assessed patients with BM are included in this review. Clinical trials often exclude patients with any BM or enroll only patients with clinically stable BM [50], limiting the ability to comprehensively assess the predictors of survival in patients with BM.

## Conclusion

In this literature review, we describe the epidemiology of patients with HER2+ metastatic or advanced BC with BM, including prognostic factors for developing BM, factors predictive of survival among patients with BM, and differences in survival and time to progression by HER2-targeting drug class based on drug mechanism of action. Prognostic factors associated with shorter TTBM included younger age, HR- status, no receipt of trastuzumab or anti-HER2 therapy, higher tumor grade, and larger tumor size. Predictors of longer OS after BM included receipt of anti-HER2 therapy or any systemic therapy, and the presence of fewer brain lesions (< 3 or a single lesion). Trastuzumab and lapatinib combination therapy after BM diagnosis was associated with longer OS after BM compared with other treatments assessed in this review. More research is needed to better understand risk factors for BM and treatments that may improve outcomes.

## Supplementary Information

**Additional file 1.**

## Data Availability

Data has been made available as electronic supplementary material.

## Abbreviations

BBB: Blood-brain barrier
BC: Breast cancer
BM: Brain metastasis
HER2: Human epidermal growth factor receptor 2
HR: Hormone receptor
OS: Overall survival
TKI: Tyrosine kinase inhibitor
TTBM: Time to first brain metastasis diagnosis
WBRT: Whole-brain radiotherapy
