# Supplementary material for: Prognostic factors of brain metastasis and survival among HER2-positive metastatic breast ﻿cancer patients: a systematic literature review
Source: BMC Cancer. 2021 Aug 28;21:967. doi: 10.1186/s12885-021-08708-5 (PMC8403419; doi:10.1186/s12885-021-08708-5)
Supplement: Supplementary file 1 — Additional file 1. [file 12885_2021_8708_MOESM1_ESM.docx]

# Supplemental Materials

Online Resource 1. Search Strategy for PubMed Database

| **Search No.** | **Search Terms** | **No. of Articles (original search)** | **No. of articles (updated search)** |
| --- | --- | --- | --- |
| **Disease** | | |  |
| #1 | "Breast Neoplasms"[Majr] OR breast neoplasm*[Title] OR breast cancer*[Title] OR breast carcinoma*[Title] OR breast tumor*[Title] OR breast tumour*[Title] OR mammary cancer*[Title] OR mammary carcinoma*[Title] OR mammary neoplasm*[Title] OR mammary tumor*[Title] OR mammary tumour*[Title] OR “cancer of the breast”[Title] OR breast malignan*[Title] OR mammary malignan*[Title] OR breast adenocarcinoma*[Title] OR mammary adenocarcinoma*[Title] | 122,969 | 307,470 |
| **Population** | | |  |
| #2 | "Neoplasm Metastasis"[Mesh] OR "Breast Neoplasms/secondary"[Mesh] OR "Neoplasm Recurrence, Local"[Mesh] OR metastasis[Title/Abstract] OR metastases[Title/Abstract] OR metastatic[Title/Abstract] OR metastasize*[Title/Abstract] OR “stage 3”[Title/Abstract] OR “stage III”[Title/Abstract] OR “stage three”[Title/Abstract] OR “stage 4”[Title/Abstract] OR “stage IV”[Title/Abstract] OR “stage four”[Title/Abstract] OR advanced[Title/Abstract] OR disseminat*[Title/Abstract] OR recur*[Title/Abstract] OR relaps*[Title/Abstract] OR incurable[Title/Abstract] OR untreated[Title/Abstract] OR unresectable[Title/Abstract] OR “non-resectable”[Title/Abstract] | 818,568 | 265,140 |
| #3 | "HER2-positive"[Title/Abstract] OR "HER2+"[Title/Abstract] OR “HER 2”[Title/Abstract] OR “human epidermal growth factor receptor 2”[Title/Abstract] | 22,558 | 6,664 |
| #4 | #1 AND #2 AND #3 | 8,523 | 2,707 |
| **Brain Metastasis** | | |  |
| #5 | #4 AND ("Brain Neoplasms"[Majr] OR brain neoplasm*[Title] OR brain tumor*[Title] OR brain tumour*[Title] OR brain cancer*[Title] OR brain malignan*[Title] OR “cancer of the brain”[Title] OR intracranial neoplasm*[Title] OR “brain metastasis”[Title] OR “brain metastases”[Title]) | 354 | 110 |
| **Incidence and Prevalence** | | |  |
| #6 | #5 AND (“Incidence”[Majr] OR “Prevalence”[Majr] OR incidence[Title/Abstract] OR prevalence[Title/Abstract]) | 64 | 21 |
| **Study Types** | | |  |
| #7 | #5 AND ("Clinical Trials as Topic"[Mesh] OR "Random Allocation"[Mesh] OR trial*[Title/Abstract] OR random*[Title/Abstract] OR “phase four”[Title/Abstract] OR “phase 4”[Title/Abstract] OR “phase IV”[Title/Abstract] OR “phase three”[Title/Abstract] OR “phase 3”[Title/Abstract] OR “phase III”[Title/Abstract] OR “phase two”[Title/Abstract] OR “phase 2”[Title/Abstract] OR “phase II”[Title/Abstract] OR RCT*[Title/Abstract] OR "Clinical Trial"[Publication Type] OR "Observational Studies as Topic"[Mesh] OR "Cohort Studies"[Mesh] OR "Retrospective Studies"[Mesh] OR "Cross-Sectional Studies"[Mesh] OR "Case-Control Studies"[Mesh] OR "Longitudinal Studies"[Mesh] OR "Registries"[Mesh] OR "Prospective Studies"[Mesh] OR "Follow-Up Studies"[Mesh] OR observation*[Title/Abstract] OR cohort[Title/Abstract] OR retrospective*[Title/Abstract] OR "cross-sectional"[Title/Abstract] OR nonrandomized[Title/Abstract] OR "non-randomized"[Title/Abstract] OR nonrandomised[Title/Abstract] OR "non-randomised"[Title/Abstract] OR “case control”[Title/Abstract] OR longitudinal[Title/Abstract] OR “real world”[Title/Abstract] OR prospective*[Title/Abstract] OR “follow up”[Title/Abstract] OR database stud*[Title/Abstract] OR database analys*[Title/Abstract] OR registry[Title/Abstract] OR registries[Title/Abstract] OR "Observational Study"[Publication Type]) | 217 | 71 |
| **Exclusions** | |  |  |
| #8 | “Animals”[Mesh] NOT “Humans”[Mesh] | 1,139,087 | 188,781 |
| #9 | "Comment”[Publication Type] OR “Letter”[Publication Type] OR “Editorial”[Publication Type] | 748,115 | 208,700 |
| #10 | negative[Title/Abstract] NOT positive[Title/Abstract] | 283,831 | 91,293 |
| **Total** | |  |  |
| #11 | (#6 OR #7) NOT (#8 OR #9 OR #10) | **202** | **65** |

*Updated search was run from 2019-Jul-1 to 2021-Jun-25

Online Resource 2. Original and Updated Search Strategies for Embase Database

| **Search No.** | **Search Terms** | **No. of Articles** |
| --- | --- | --- |
| **Original Search Strategy for Embase (January 1, 2009 to July 30, 2019)** | | |
| **Disease** | | |
| #1 | ('breast tumor'/exp/mj OR ((breast NEXT/1 neoplasm*):ti) OR ((breast NEXT/1 cancer*):ti) OR ((breast NEXT/1 carcinoma*):ti) OR ((breast NEXT/1 tumor*):ti) OR ((breast NEXT/1 tumour*):ti) OR ((mammary NEXT/1 cancer*):ti) OR ((mammary NEXT/1 carcinoma*):ti) OR ((mammary NEXT/1 neoplasm*):ti) OR ((mammary NEXT/1 tumor*):ti) OR ((mammary NEXT/1 tumour*):ti) OR 'cancer of the breast':ti OR ((breast NEXT/1 malignan*):ti) OR ((mammary NEXT/1 malignan*):ti) OR ((breast NEXT/1 adenocarcinoma*):ti) OR ((mammary NEXT/1 adenocarcinoma*):ti)) AND [english]/lim AND [embase]/lim AND [2009-2019]/py | 172,858 |
| **Population** | | |
| #2 | ('metastasis'/exp OR 'tumor recurrence'/exp OR metastasis:ti,ab OR metastases:ti,ab OR metastatic:ti,ab OR metastasize*:ti,ab OR 'stage 3':ti,ab OR 'stage iii':ti,ab OR 'stage three':ti,ab OR 'stage 4':ti,ab OR 'stage iv':ti,ab OR 'stage four':ti,ab OR advanced:ti,ab OR disseminat*:ti,ab OR recur*:ti,ab OR relaps*:ti,ab OR incurable:ti,ab OR untreated:ti,ab OR unresectable:ti,ab OR 'non-resectable':ti,ab) AND [english]/lim AND [embase]/lim AND [2009-2019]/py | 1,356,341 |
| #3 | ('her2-positive':ti,ab OR 'her2+':ti,ab OR 'her 2':ti,ab OR 'human epidermal growth factor receptor 2':ti,ab) AND [english]/lim AND [embase]/lim AND [2009-2019]/py | 46,297 |
| #4 | #1 AND #2 AND #3 | 20,265 |
| **Brain Metastasis** | | |
| #5 | #4 AND ('brain tumor'/exp/mj OR ((brain NEXT/1 neoplasm*):ti) OR ((brain NEXT/1 tumor*):ti) OR ((brain NEXT/1 tumour*):ti) OR ((brain NEXT/1 cancer*):ti) OR ((brain NEXT/1 malignan*):ti) OR 'cancer of the brain':ti OR ((intracranial NEXT/1 neoplasm*):ti) OR 'brain metastasis':ti OR 'brain metastases':ti) AND [english]/lim AND [embase]/lim AND [2009-2019]/py | 733 |
| **Incidence and Prevalence** | | |
| #6 | #5 AND ('incidence'/exp/mj OR 'prevalence'/exp/mj OR incidence:ti,ab OR prevalence:ti,ab) AND [english]/lim AND [embase]/lim AND [2009-2019]/py | 167 |
| **Study Types** | | |
| #7 | #5 AND ('clinical trial (topic)'/exp OR 'randomization'/exp OR trial*:ti,ab OR random*:ti,ab OR 'phase four':ti,ab OR 'phase 4':ti,ab OR 'phase iv':ti,ab OR 'phase three':ti,ab OR 'phase 3':ti,ab OR 'phase iii':ti,ab OR 'phase two':ti,ab OR 'phase 2':ti,ab OR 'phase ii':ti,ab OR rct*:ti,ab OR 'clinical trial'/exp OR 'observational study'/exp OR 'cohort analysis'/exp OR 'retrospective study'/exp OR 'cross-sectional study'/exp OR 'case control study'/exp OR 'longitudinal study'/exp OR 'register'/exp OR 'prospective study'/exp OR 'follow up'/exp OR observation*:ti,ab OR cohort:ti,ab OR retrospective*:ti,ab OR 'cross-sectional':ti,ab OR nonrandomized:ti,ab OR 'non-randomized':ti,ab OR nonrandomised:ti,ab OR 'non-randomised':ti,ab OR 'case control':ti,ab OR longitudinal:ti,ab OR 'real world':ti,ab OR prospective*:ti,ab OR 'follow up':ti,ab OR ((database NEXT/1 stud*):ti,ab) OR ((database NEXT/1 analys*):ti,ab) OR registry:ti,ab OR registries:ti,ab) AND [english]/lim AND [embase]/lim AND [2009-2019]/py | 487 |
| **Exclusions** | |  |
| #8 | 'animal'/exp NOT 'human'/exp AND [english]/lim AND [embase]/lim AND [2009-2019]/py | 1,323,564 |
| #9 | (comment*:ti OR letter:it OR editorial:it OR 'conference abstract':it OR 'conference paper':it) AND [english]/lim AND [embase]/lim AND [2009-2019]/py | 4,145,082 |
| #10 | negative:ti,ab NOT positive:ti,ab AND [english]/lim AND [embase]/lim AND [2009-2019]/py | 380,840 |
| **Total** | |  |
| #11 | (#6 OR #7) NOT (#8 OR #9 OR #10) | **179** |
| **Updated Search Strategy for Embase (through June 24, 2021)** | | |
| **Search No.** | **Search Terms** | **No. of Articles** |
| **Disease** | | |
| 1 | exp breast cancer/ | 488,183 |
| 2 | ((breast adj1 neoplasm*) or (breast adj1 cancer*) or (breast adj1 carcinoma*) or (breast adj1 tumor*) or (breast adj1 tumour*) or (mammary adj1 cancer*) or (mammary adj1 carcinoma*) or (mammary adj1 neoplasm*) or (mammary adj1 tumor*) or (mammary adj1 tumour*) or cancer of the breast or (breast adj1 malignan*) or (mammary adj1 malignan*) or (breast adj1 adenocarcinoma*) or (mammary adj1 adenocarcinoma*)).ti. | 300,391 |
| 3 | 1 or 2 | 535,261 |
| **Population** | | |
| 4 | exp metastasis/ or exp tumor recurrence/ | 702,960 |
| 5 | (metastasis or metastases or metastatic or metastasize* or stage 3 or stage iii or stage three or stage 4 or stage iv or stage four or advanced or disseminat* or recur* or relaps* or incurable or untreated or unresectable or non-resectable).ti,ab. | 2,710,863 |
| 6 | 4 or 5 | 2,836,526 |
| 7 | (her2-positive or her2+ or her 2 or human epidermal growth factor receptor 2).ti,ab. | 70,551 |
| 8 | 3 and 6 and 7 | 32,535 |
| **Brain Metastasis** | | |
| 9 | exp brain tumor/ | 172,838 |
| 10 | ((brain adj1 neoplasm*) or (brain adj1 tumor*) or (brain adj1 tumour*) or (brain adj1 cancer*) or (brain adj1 malignan*) or cancer of the brain or (intracranial adj1 neoplasm*) or brain metastasis or brain metastases).ti. | 35,069 |
| 11 | 9 or 10 | 175,643 |
| 12 | 8 and 11 | 2,153 |
| **Incidence and Prevalence** | | |
| 13 | exp incidence/ or exp prevalence/ | 1,292,413 |
| 14 | (incidence or prevalence).ti,ab. | 1,958,741 |
| 15 | 13 or 14 | 2,280,683 |
| 16 | 12 and 15 | 398 |
| **Study Types** | | |
| 17 | exp clinical trial as topic/ or exp randomization/ or exp clinical trial/ or exp observational study/ or exp cohort analysis/ or exp retrospective study/ or exp cross-sectional study/ or case control study/ or exp longitudinal study/ or exp register/ or exp prospective study/ | 4,232,073 |
| 18 | (trial* or random* or phase four or phase 4 or phase iv or phase three or phase 3 or phase iii or phase two or phase 2 or phase ii or rct* or observation* or cohort or retrospective* or cross-sectional or nonrandomized or non-randomized or nonrandomized or non-randomised or case control or longitudinal or real world or prospective* or follow-up or (database adj1 stud*) or (database adj1 analys*) or registry or registries).ti,ab. | 7,305,091 |
| 19 | 17 or 18 | 8,162,583 |
| 20 | 12 and 19 | 1,444 |
| **Exclusions** | | |
| 21 | exp animals/ | 26,421,720 |
| 22 | exp humans/ | 21,996,042 |
| 23 | 21 not 22 | 4,425,678 |
| 24 | (commentary or letter or editorial or conference abstract or conference paper).pt. | 6,707,915 |
| 25 | negative.ti,ab. | 1,566,422 |
| 26 | positive.ti,ab. | 2,231,183 |
| 27 | 25 not 26 | 851,569 |
| 28 | (16 or 20) not (23 or 24 or 27) | 647 |
| 29 | limit 28 to english | 621 |
| **Total** | | |
| 30 | limit 29 to dc=20190701-20210625 | **151** |

Online Resource 3. Study and Patient Characteristics

| Citation | Study Population | Country | Study Observation Period (Date Range) | Overall Sample Size, n | HER2+ Patients | | | |
| --- | --- | --- | --- | --- | --- | --- | --- | --- |
|  |  |  |  |  | Sample Size, n (%) | BM, n (%) | HR+, n (%) | Age, Median (Mean), y^a^ |
| Ahn et al., 2013 [[12](#_ENREF_12)] | Patients with mBC with BM treated with trastuzumab | Korea | 2000-2011 | 189 | 86 (45.5) | 86 (100) | 37 (43.0) | 45-46^b^ |
| Anders et al., 2011 [[10](#_ENREF_10)] | Patients with mBC with CNS metastasis treated with chemotherapy | US | 1988-2008 | 119 | 39 (32.8) | 39 (100) | 21 (53.8) | 43-49 |
| Berghoff et al., 2012 [[24](#_ENREF_24)] | Patients treated for symptomatic BM from BC | Austria | 1996-2010 | 213 | 124 (58.2) | 124 (100) | NR | 50^c^ |
| Braccini et al., 2013 [[29](#_ENREF_29)] | Patients with BC with BM | France | 1995-2010 | 250 | 109 (43.6) | 109 (100) | ER+: 40 (36.7)  PR+:30 (27.5) | 51 |
| Brufsky et al., 2011 [[25](#_ENREF_25)] | Patients with HER2+ mBC | US | 2003-2006 | 1,012 | 1,012 (100) | 377 (37.3) | 167 (44.3) | < 50: 162 (43.0)^d^  50-64: 157 (41.6)^d^  ≥ 65: 58 (15.4)^d^ |
| Duchnowska et al., 2012 [[15](#_ENREF_15)] | Patients with HER2+ mBC administered at least 1 dose of trastuzumab-based therapy | Poland | 2000-2010 | 142 | 142 (100) | 49 (34.5) | ER+:55 (38.7)  PR+:43 (30.3) | 53^b^ |
| Duchnowska et al., 2009 [[16](#_ENREF_16)] | Patients with HER2+ mBC | Poland | 1993-2007 | 264 | 264 (100) | 103 (39.0) | ER+: 96 (36.4)  PR+: 69 (26.1) | (49) |
| Duchnowska et al., 2015 [[17](#_ENREF_17)] | Patients with HER2+ mBC | Poland, Serbia, US | Cohort A (discovery): 2006-2008^e^ | 84 | 84 (100) | 48 (57.1) | ER+: 31 (36.9)  PR+: 24 (28.6) | (50) |
|  |  |  | Cohort B (validation): 2008-2010^e^ | 75 | 75 (100) | 41 (54.7) | ER+: 32 (43)  PR+: 21 (28) | (52) |
| Gori et al., 2019 [[18](#_ENREF_18)] | Patients with HER2+ BC and BM | Italy | 2005-2014 | 154 | 154 (100) | 154 (100) | 60 (39) | 53 |
| Hayashi et al., 2015 [[19](#_ENREF_19)] | Patients with HER2+ BC diagnosed with BM | Japan | 2001-2012 | 432 | 432 (100) | 432 (100) | ER+: 162 (37.5) | 54 |
| Heitz et al., 2009 [[23](#_ENREF_23)] | Patients with primary invasive BC | Germany | 1998-2006 | 2,441 | 245 (10.1) | 19 (7.8) | 150 (61.2) | > 50: 142 (60.0)^d^  ≤ 50: 103 (40.0)^d^ |
| Jang et al., 2011 [[27](#_ENREF_27)] | Patients with BC and BM | Korea | 1990-2006 | 137 | 69 (50.4) | 69 (100) | NR | 45 |
| Kaplan et al., 2012 [[26](#_ENREF_26)] | Patients with BC and BM | Turkey | 2001-2011 | 422 | 215 (51.0) | 215 (100) | 102 (24.2) | 43-45 |
| Kuba et al., 2014 [[28](#_ENREF_28)] | Patients with BC and BM | Japan | 2001-2010 | 65 | 26 (40.0) | 26 (100) | NR | 53.5 |
| Martin et al., 2017 [[36](#_ENREF_36)] | Patients newly diagnosed with BC with BM | US | 2010-2013 | 968 | 242 (25.0) | 242 (100) | 136 (56.2) | NR |
| Maurer et al., 2018 [[20](#_ENREF_20)] | Patients with HER2+ BC | Belgium | 2000-2014 | 483 | 483 (100) | 52 (10.8) | 343 (71.0) | 49.7-53.9 |
| Morikawa et al., 2018 [[21](#_ENREF_21)] | Patients with HER2+ mBC with BM, treated via SRS or WBRT as initial primary/definitive treatment for BM | US | 2001-2011 | 100 | 100 (100) | 100 (100) | 61 (61.0) | 54 |
| Mounsey et al., 2018 [[14](#_ENREF_14)] | Patients with HER2+ BC with BM | US | 1998-2015 | 123 | 123 (100) | 123 (100) | 54 (43.9) | 51 |
| Niwinska et al., 2010 [[30](#_ENREF_30)] | Patients with BC with BM | Poland | 2003-2006 | 222 | 109 (49.1) | 109 (100) | 48 (44.0) | 48 |
| Sperduto et al., 2013 [[35](#_ENREF_35)] | Women with BM from BC with adequate data to define tumor subtype | US, Canada | 1993-2010 | 383 | 217 (56.7) | 217 (100) | 98 (45.2) | 53-55 |
| Witzel et al., 2018 [[11](#_ENREF_11)] | Patients with BC with BM | Germany | 2000-2016 | 1,712 | 732 (47.8) | 732 (100) | NR | 51^c^ |
| Yap et al., 2012 [[13](#_ENREF_13)] | Consecutive female patients with HER2+ BC diagnosed with BM | Indonesia, Korea, Malaysia, Philippines, Singapore, Thailand | 2006-2008 | 280 | 280 (100) | 280 (100) | 119 (42.5) | 48 |
| Zhang, Q et al., 2016 [[22](#_ENREF_22)] | Consecutive patients with HER2+ BC with BM undergoing WBRT | China | 2006-2012 | 60 | 60 (100) | 60 (100) | ER+: 31 (51.7) | 46 |
| Anwar et al., 2021 [50] | Patients with HER2+ mBC treated with pyrotinib | China | 2018-2020 | 168 | 168 (100) | 39 (23.2) | 92 (54.8) | <50: 82 (48.8)^d^  ≥50: 86 (51.2)^d^ |
| Bergen et al., 2021 [49] | Patients with HER2+ BC with BM | Austria | 1990-2019 | 252 | 252 (100) | 252 (100) | ER+: 109 (43.3) | 49.5 |

BC = breast cancer; BM = brain metastasis; CNS = central nervous system; ER = estrogen receptor; HER2 = human epidermal growth factor receptor 2; HR = hormone receptor; mBC = metastatic breast cancer; NR = not reported; PR = progesterone receptor; SRS = stereotactic radiosurgery; US = United States; WBRT = whole-brain radiotherapy.

^a^ At breast cancer diagnosis.

^b^ At distant metastasis or disease progression.

^c^ Among the overall population irrespective of HER2 status; age is not reported for HER2+ patients specifically.

^d^ Values are n (%) instead of median (or mean).

^e^ The period the material was collected for evaluation. Patients were treated during the period from 1993 to 2010.

Online Resource 4. PRISMA Checklist.

| **Section/topic** | **#** | **Checklist item** | **Reported on page #** |
| --- | --- | --- | --- |
| **TITLE** | | |  |
| Title | 1 | Identify the report as a systematic review, meta-analysis, or both. | 1 |
| **ABSTRACT** | | |  |
| Structured summary | 2 | Provide a structured summary including, as applicable: background; objectives; data sources; study eligibility criteria, participants, and interventions; study appraisal and synthesis methods; results; limitations; conclusions and implications of key findings; systematic review registration number. | 1 |
| **INTRODUCTION** | | |  |
| Rationale | 3 | Describe the rationale for the review in the context of what is already known. | 2 |
| Objectives | 4 | Provide an explicit statement of questions being addressed with reference to participants, interventions, comparisons, outcomes, and study design (PICOS). | 2 |
| **METHODS** | | |  |
| Protocol and registration | 5 | Indicate if a review protocol exists, if and where it can be accessed (e.g., Web address), and, if available, provide registration information including registration number. | N/A |
| Eligibility criteria | 6 | Specify study characteristics (e.g., PICOS, length of follow-up) and report characteristics (e.g., years considered, language, publication status) used as criteria for eligibility, giving rationale. | 2-3, Table 1 |
| Information sources | 7 | Describe all information sources (e.g., databases with dates of coverage, contact with study authors to identify additional studies) in the search and date last searched. | 2 |
| Search | 8 | Present full electronic search strategy for at least one database, including any limits used, such that it could be repeated. | Online Resource 1-2 |
| Study selection | 9 | State the process for selecting studies (i.e., screening, eligibility, included in systematic review, and, if applicable, included in the meta-analysis). | 3 |
| Data collection process | 10 | Describe method of data extraction from reports (e.g., piloted forms, independently, in duplicate) and any processes for obtaining and confirming data from investigators. | 3 |
| Data items | 11 | List and define all variables for which data were sought (e.g., PICOS, funding sources) and any assumptions and simplifications made. | 3 |
| Risk of bias in individual studies | 12 | Describe methods used for assessing risk of bias of individual studies (including specification of whether this was done at the study or outcome level), and how this information is to be used in any data synthesis. | 3, 8 |
| Summary measures | 13 | State the principal summary measures (e.g., risk ratio, difference in means). | Table 1 |
| Synthesis of results | 14 | Describe the methods of handling data and combining results of studies, if done, including measures of consistency (e.g., I^2^) for each meta-analysis. | N/A |

Page 1 of 2

| **Section/topic** | **#** | **Checklist item** | **Reported on page #** |
| --- | --- | --- | --- |
| Risk of bias across studies | 15 | Specify any assessment of risk of bias that may affect the cumulative evidence (e.g., publication bias, selective reporting within studies). | 8 |
| Additional analyses | 16 | Describe methods of additional analyses (e.g., sensitivity or subgroup analyses, meta-regression), if done, indicating which were pre-specified. | N/A |
| **RESULTS** | | |  |
| Study selection | 17 | Give numbers of studies screened, assessed for eligibility, and included in the review, with reasons for exclusions at each stage, ideally with a flow diagram. | Figure 1 |
| Study characteristics | 18 | For each study, present characteristics for which data were extracted (e.g., study size, PICOS, follow-up period) and provide the citations. | Online Resource 3 |
| Risk of bias within studies | 19 | Present data on risk of bias of each study and, if available, any outcome level assessment (see item 12). | 8, Table 5 |
| Results of individual studies | 20 | For all outcomes considered (benefits or harms), present, for each study: (a) simple summary data for each intervention group (b) effect estimates and confidence intervals, ideally with a forest plot. | 4-8, Tables 2-4 |
| Synthesis of results | 21 | Present results of each meta-analysis done, including confidence intervals and measures of consistency. | N/A |
| Risk of bias across studies | 22 | Present results of any assessment of risk of bias across studies (see Item 15). | 8, Table 5 |
| Additional analysis | 23 | Give results of additional analyses, if done (e.g., sensitivity or subgroup analyses, meta-regression [see Item 16]). | N/A |
| **DISCUSSION** | | |  |
| Summary of evidence | 24 | Summarize the main findings including the strength of evidence for each main outcome; consider their relevance to key groups (e.g., healthcare providers, users, and policy makers). | 8-9 |
| Limitations | 25 | Discuss limitations at study and outcome level (e.g., risk of bias), and at review-level (e.g., incomplete retrieval of identified research, reporting bias). | 9-10 |
| Conclusions | 26 | Provide a general interpretation of the results in the context of other evidence, and implications for future research. | 10 |
| **FUNDING** | | |  |
| Funding | 27 | Describe sources of funding for the systematic review and other support (e.g., supply of data); role of funders for the systematic review. | 11 |

*From:*  Moher D, Liberati A, Tetzlaff J, Altman DG, The PRISMA Group (2009). Preferred Reporting Items for Systematic Reviews and Meta-Analyses: The PRISMA Statement. PLoS Med 6(7): e1000097. doi:10.1371/journal.pmed1000097
